# 9-(4-Bromo­phenoxy­carbon­yl)-10-methyl­acridinium trifluoro­methane­sulfonate

**DOI:** 10.1107/S1600536810016296

**Published:** 2010-05-12

**Authors:** Damian Trzybiński, Karol Krzymiński, Artur Sikorski, Jerzy Błażejowski

**Affiliations:** a Faculty of Chemistry, University of Gdańsk, J. Sobieskiego 18, 80-952 Gdańsk, Poland

## Abstract

In the crystal structure of the title compound, C_21_H_15_BrNO_2_^+^·CF_3_SO_3_^−^, the cations form inversion dimers through π–π inter­actions between the acridine ring systems. These dimers are further linked by C—H⋯π and C—Br⋯π inter­actions. The cations and anions are connected by multidirectional C—H⋯O and C—F⋯π inter­actions. The acridine and benzene ring systems are oriented at 10.8 (1)°. The carboxyl group is twisted at an angle of 85.2 (1)° relative to the acridine skeleton. The mean planes of adjacent acridine units are parallel or almost parallel [inclined at an angle of 1.4 (1)°] in the crystal structure.

## Related literature

For background to the chemiluminogenic properties of 9-phenoxy­carbonyl-10-methyl­acridinium trifluoro­methane­sulf­onates, see: Adamczyk & Mattingly (2002 ▶); King *et al.* (2007 ▶); Rak *et al.* (1999 ▶); Roda *et al.* (2003 ▶); Zomer & Jacquemijns (2001 ▶). For related structures, see: Sikorski *et al.* (2005*a* ▶,*b* ▶). For inter­molecular inter­actions, see: Bianchi *et al.* (2004 ▶); Dorn *et al.* (2005 ▶); Hunter *et al.* (2001 ▶); Novoa *et al.* (2006 ▶); Seo *et al.* (2009 ▶); Takahashi *et al.* (2001 ▶). For the synthesis, see: Sato (1996 ▶); Sikorski *et al.* (2005*a* ▶,*b* ▶).
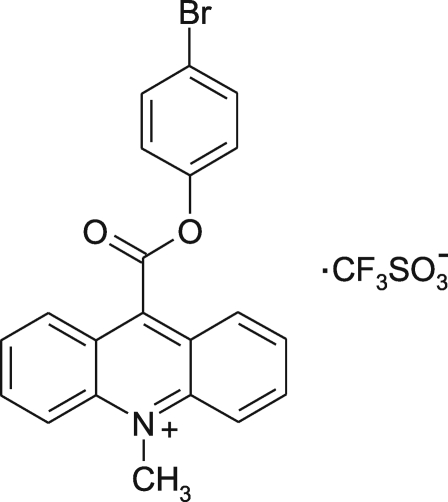


## Experimental

### 

#### Crystal data

C_21_H_15_BrNO_2_^+^·CF_3_O_3_S^−^
*M* *_r_* = 542.32
Monoclinic, 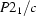

*a* = 9.5755 (2) Å
*b* = 20.4912 (7) Å
*c* = 11.6241 (5) Å
β = 104.011 (3)°
*V* = 2212.95 (13) Å^3^
*Z* = 4
Mo *K*α radiation
μ = 2.01 mm^−1^
*T* = 295 K
0.37 × 0.15 × 0.05 mm

#### Data collection

Oxford Diffraction Gemini R Ultra Ruby CCD diffractometer
Absorption correction: multi-scan (*CrysAlis RED*; Oxford Diffraction, 2008 ▶) *T*_min_ = 0.77, *T*_max_ = 0.92
50472 measured reflections
3910 independent reflections
2200 reflections with *I* > 2σ(*I*)
*R*_int_ = 0.048

#### Refinement

*R*[*F* ^2^ > 2σ(*F* ^2^)] = 0.039
*wR*(*F* ^2^) = 0.112
*S* = 0.98
3910 reflections
299 parameters
H-atom parameters constrained
Δρ_max_ = 0.56 e Å^−3^
Δρ_min_ = −0.62 e Å^−3^

### 

Data collection: *CrysAlis CCD* (Oxford Diffraction, 2008 ▶); cell refinement: *CrysAlis RED* (Oxford Diffraction, 2008 ▶); data reduction: *CrysAlis RED*; program(s) used to solve structure: *SHELXS97* (Sheldrick, 2008 ▶); program(s) used to refine structure: *SHELXL97* (Sheldrick, 2008 ▶); molecular graphics: *ORTEP-3* (Farrugia, 1997 ▶); software used to prepare material for publication: *SHELXL97* and *PLATON* (Spek, 2009 ▶).

## Supplementary Material

Crystal structure: contains datablocks global, I. DOI: 10.1107/S1600536810016296/om2334sup1.cif

Structure factors: contains datablocks I. DOI: 10.1107/S1600536810016296/om2334Isup2.hkl

Additional supplementary materials:  crystallographic information; 3D view; checkCIF report

## Figures and Tables

**Table 1:** Hydrogen-bond geometry (Å, °) *Cg*4 is the centroid of the C18–C23 ring.

*D*—H⋯*A*	*D*—H	H⋯*A*	*D*⋯*A*	*D*—H⋯*A*
C2—H2⋯O27^i^	0.93	2.59	3.361 (5)	141
C4—H4⋯O28^ii^	0.93	2.50	3.365 (4)	155
C20—H20⋯O27	0.93	2.50	3.176 (4)	130
C25—H25*A*⋯*Cg*4^iii^	0.96	2.81	3.569 (4)	136
C25—H25*B*⋯O28^ii^	0.96	2.53	3.472 (5)	167

Symmetry codes: (i) 
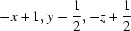
; (ii) 
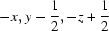
; (iii) 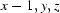.

**Table 2:** C–Br⋯π and C–F⋯π inter­actions (Å,°) *Cg*1, *Cg*3 and *Cg*4 are the centroids of the C9/N10/C11–C14, C5–C8/C13/C14 and C18–C23 rings, respectively.

*X*	*I*	*J*	*I*⋯*J*	*X*⋯*J*	*X*–*I*⋯*J*
C21	Br24	*Cg*1^iv^	3.958 (2)	4.158 (3)	82.3 (1)
C21	Br24	*Cg*3^iv^	3.937 (2)	4.235 (4)	85.4 (2)
C30	F31	*Cg*4^v^	3.212 (4)	4.305 (5)	137.5 (3)

Symmetry codes: (iv) 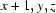; (v) 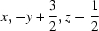.

**Table 3:** π–π inter­actions (Å,°) *Cg*1 and *Cg*2 are the centroids of the C9/N10/C11–C14 and C1–C4/C11/C12 rings, respectively. *CgI*⋯*CgJ* is the distance between ring centroids. The dihedral angle is that between the planes of the rings *I* and *J. CgI*_Perp is the perpendicular distance of *CgI* from ring *J. CgI*_Offset is the distance between *CgI* and perpendicular projection of *CgJ* on ring *I*.

*I*	*J*	*CgI*⋯*CgJ*	Dihedral angle	*CgI*_Perp	*CgI*_Offset
1	2^vi^	3.650 (2)	2.82 (16)	3.623 (2)	0.444 (2)

Symmetry code: (vi) 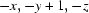.

**Fig. 1.**
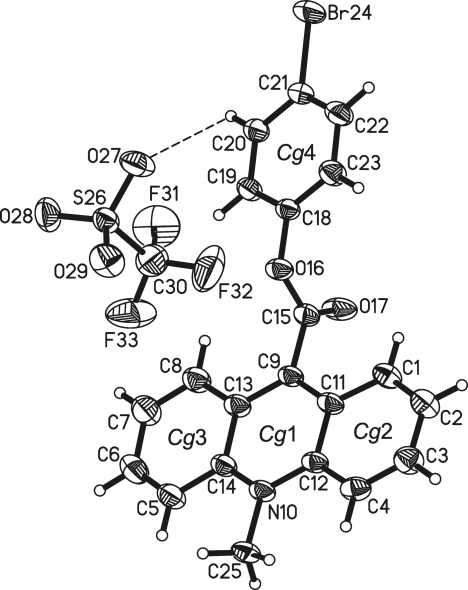
The molecular structure of the title compound showing the atom-labeling scheme. Displacement ellipsoids are drawn at the 25% probability level and H atoms are shown as small spheres of arbitrary radius. Cg1, Cg2, Cg3 and Cg4 denote the ring centroids. The C–H···O hydrogen bond is represented by a dashed line.

**Fig. 2.**
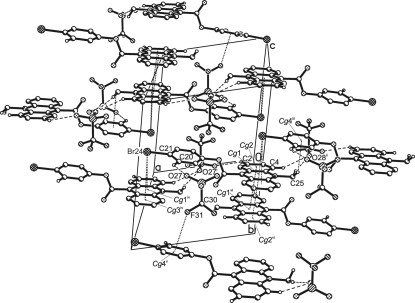
The arrangement of the ions in the crystal structure. The C–H···O interactions are represented by dashed lines, the C–H···π, C–F···π, C–Br–π and π–π contacts by dotted lines. H atoms not involved in interactions have been omitted. [Symmetry codes: (i) –x + 1, y – 1/2, –z + 1/2; (ii) –x, y – 1/2, –z + 1/2; (iii) x – 1, y, z; (iv) x + 1, y, z; (v) x, –y + 3/2, z – 1/2; (vi) –x, –y + 1, –z.]

## supplementary crystallographic information

### Comment

The cations of 9-(phenoxycarbonyl)-10-methylacridinium salts react efficiently with H_2_O_2_ in alkaline media producing light (Zomer & Jacquemijns, 2001; Adamczyk & Mattingly, 2002). This effect means that the compounds can serve as chemiluminescent indicators or as chemiluminogenic fragments of chemiluminescent labels in assays of biologically and environmentally important entities such as antigens, antibodies, enzymes or DNA fragments (Zomer & Jacquemijns, 2001; Adamczyk & Mattingly, 2002; Roda *et al.* , 2003; King *et al.*, 2007). The chemiluminogenic features of the compounds depend on the structure of the cations, particularly the phenoxycarbonyl fragment which is removed during their oxidation leading to electronically excited 10-methyl-9-acridinone molecules (Rak *et al.*, 1999; Zomer & Jacquemijns, 2001). It has been found that the efficiency of chemiluminescence – crucial for analytical applications – is influenced by the constitution of the phenyl fragment (Zomer & Jacquemijns, 2001). This prompted us to synthesize and investigate derivatives substituted in this latter fragment. In this paper, a continuation of a series on bromo-substituted derivatives (Sikorski *et al.*, 2005*a*), we present the structure of the title compound.

In the cation of the title compound (Fig. 1), the bond lengths and angles characterizing the geometry of the acridinium moiety are typical of acridine-based derivatives (Sikorski *et al.*, 2005*a*,*b*). With respective average deviations from planarity of 0.0442 (3) Å and 0.0046 (3) Å, the acridine and benzene ring systems are oriented at 10.8 (1)°. The carboxyl group is twisted at an angle of 85.2 (1)° relative to the acridine skeleton. The mean planes of the adjacent acridine moieties are parallel (remain at an angle of 0.0 (1)°) or almost parallel (remain at an angle of 1.4 (1)°) in the lattice.

In the crystal structure, the inversely oriented cations form dimers through π–π interactions involving acridine moieties (Table 3, Fig. 2). These dimers are further linked by C–H···π (Table 1, Fig. 2) and C–Br···π (Table 2, Fig. 2) interactions. The cations and anions are connected by multidirectional C–H···O (Table 1, Fig. 2) and C–F···π (Table 2, Fig. 2) interactions. The C–H···O interactions are of the hydrogen bond type (Bianchi *et al.*, 2004; Novoa *et al.*, 2006). The C–H···π interactions should be of an attractive nature (Takahashi *et al.*, 2001), like the C–F···π (Dorn *et al.*, 2005) and π–π (Hunter *et al.*, 2001) interactions. The C–Br···π interactions have been reported by others (Seo *et al.*, 2009). The crystal structure is stabilized by a network of these short-range specific interactions and by long-range electrostatic interactions between ions.

### Experimental

The title compound was obtained by treating 4-bromophenyl acridine-9-carboxylate [synthesized by heating acridine-9-carboxylic acid with a tenfold molar excess of thionyl chloride and reacting the product thus obtained with an equimolar amount of 4-bromophenol (Sato, 1996; Sikorski *et al.*, 2005*b*)] with a fivefold molar excess of methyl trifluoromethanesulfonate dissolved in dichloromethane (Sikorski *et al.*, 2005*a*). The crude 9-(4-bromophenoxycarbonyl)-10-methylacridinium trifluoromethanesulfonate was dissolved in a small amount of ethanol, filtered and precipitated with a 25 v/v excess of diethyl ether. Yellow crystals suitable for X-ray investigations were grown from anhydrous ethanol (m.p. 504 - 505 K).

### Refinement

H atoms were positioned geometrically, with C—H = 0.93 Å and 0.96 Å for the aromatic and methyl H atoms, respectively, and constrained to ride on their parent atoms with *U*_iso_(H) = *xU*_eq_(C), where *x* = 1.2 for the aromatic and *x* = 1.5 for the methyl H atoms.

### Crystal data

**Table:**

C_21_H_15_BrNO_2_^+^·CF_3_O_3_S^−^	*F*(000) = 1088
*M**_r_* = 542.32	*D*_x_ = 1.628 Mg m^−^^3^
Monoclinic, *P*2_1_/*c*	Mo *K*α radiation, λ = 0.71073 Å
Hall symbol: -P 2ybc	Cell parameters from 14728 reflections
*a* = 9.5755 (2) Å	θ = 3.0–29.2°
*b* = 20.4912 (7) Å	µ = 2.01 mm^−^^1^
*c* = 11.6241 (5) Å	*T* = 295 K
β = 104.011 (3)°	Plate, yellow
*V* = 2212.95 (13) Å^3^	0.37 × 0.15 × 0.05 mm
*Z* = 4	

### Data collection

**Table:**

Oxford Diffraction Gemini R Ultra Ruby CCD diffractometer	3910 independent reflections
Radiation source: enhanced (Mo) X-ray Source	2200 reflections with *I* > 2σ(*I*)
graphite	*R*_int_ = 0.048
Detector resolution: 10.4002 pixels mm^-1^	θ_max_ = 25.1°, θ_min_ = 3.0°
ω scans	*h* = −11→11
Absorption correction: multi-scan (*CrysAlis RED*; Oxford Diffraction, 2008)	*k* = −24→24
*T*_min_ = 0.77, *T*_max_ = 0.92	*l* = −13→13
50472 measured reflections	

### Refinement

**Table:**

Refinement on *F*^2^	Primary atom site location: structure-invariant direct methods
Least-squares matrix: full	Secondary atom site location: difference Fourier map
*R*[*F*^2^ > 2σ(*F*^2^)] = 0.039	Hydrogen site location: inferred from neighbouring sites
*wR*(*F*^2^) = 0.112	H-atom parameters constrained
*S* = 0.98	*w* = 1/[σ^2^(*F*_o_^2^) + (0.0642*P*)^2^] where *P* = (*F*_o_^2^ + 2*F*_c_^2^)/3
3910 reflections	(Δ/σ)_max_ = 0.001
299 parameters	Δρ_max_ = 0.56 e Å^−^^3^
0 restraints	Δρ_min_ = −0.62 e Å^−^^3^

### Special details

**Table:**

Geometry. All esds (except the esd in the dihedral angle between two l.s. planes) are estimated using the full covariance matrix. The cell esds are taken into account individually in the estimation of esds in distances, angles and torsion angles; correlations between esds in cell parameters are only used when they are defined by crystal symmetry. An approximate (isotropic) treatment of cell esds is used for estimating esds involving l.s. planes.

### Fractional atomic coordinates and isotropic or equivalent isotropic displacement parameters (Å^2^)

**Table:**

	*x*	*y*	*z*	*U*_iso_*/*U*_eq_	
C1	0.1494 (3)	0.43387 (19)	0.1754 (3)	0.0703 (10)	
H1	0.2493	0.4359	0.1910	0.084*	
C2	0.0842 (4)	0.3755 (2)	0.1601 (4)	0.0794 (11)	
H2	0.1387	0.3376	0.1642	0.095*	
C3	−0.0654 (4)	0.3715 (2)	0.1382 (3)	0.0787 (11)	
H3	−0.1092	0.3307	0.1283	0.094*	
C4	−0.1483 (3)	0.4255 (2)	0.1309 (3)	0.0684 (10)	
H4	−0.2477	0.4213	0.1171	0.082*	
C5	−0.1859 (4)	0.6625 (2)	0.1220 (3)	0.0773 (11)	
H5	−0.2858	0.6599	0.1022	0.093*	
C6	−0.1200 (4)	0.7217 (2)	0.1332 (4)	0.0902 (12)	
H6	−0.1766	0.7591	0.1205	0.108*	
C7	0.0303 (4)	0.7284 (2)	0.1631 (4)	0.0896 (12)	
H7	0.0725	0.7696	0.1708	0.108*	
C8	0.1126 (4)	0.67394 (19)	0.1808 (3)	0.0734 (10)	
H8	0.2123	0.6781	0.2009	0.088*	
C9	0.1328 (3)	0.55402 (17)	0.1829 (3)	0.0544 (8)	
N10	−0.1650 (2)	0.54462 (15)	0.1304 (2)	0.0582 (7)	
C11	0.0688 (3)	0.49303 (17)	0.1682 (3)	0.0574 (9)	
C12	−0.0853 (3)	0.48819 (17)	0.1439 (3)	0.0553 (8)	
C13	0.0508 (3)	0.61081 (17)	0.1694 (3)	0.0601 (9)	
C14	−0.1030 (3)	0.60523 (18)	0.1404 (3)	0.0596 (9)	
C15	0.2939 (3)	0.56004 (16)	0.2072 (3)	0.0573 (8)	
O16	0.35203 (19)	0.56616 (11)	0.32302 (19)	0.0620 (6)	
O17	0.3586 (2)	0.55888 (15)	0.1321 (2)	0.0855 (8)	
C18	0.5032 (3)	0.57634 (17)	0.3564 (3)	0.0521 (8)	
C19	0.5556 (3)	0.63811 (17)	0.3571 (3)	0.0606 (9)	
H19	0.4934	0.6730	0.3332	0.073*	
C20	0.7017 (3)	0.64830 (17)	0.3934 (3)	0.0647 (9)	
H20	0.7396	0.6901	0.3933	0.078*	
C21	0.7905 (3)	0.59614 (18)	0.4296 (3)	0.0639 (9)	
C22	0.7375 (4)	0.5340 (2)	0.4306 (3)	0.0796 (11)	
H22	0.7995	0.4992	0.4566	0.095*	
C23	0.5906 (4)	0.52369 (18)	0.3924 (3)	0.0713 (10)	
H23	0.5522	0.4819	0.3913	0.086*	
Br24	0.99257 (4)	0.61111 (2)	0.48030 (5)	0.1038 (2)	
C25	−0.3252 (3)	0.5406 (2)	0.1034 (3)	0.0775 (11)	
H25A	−0.3609	0.5716	0.1511	0.116*	
H25B	−0.3533	0.4974	0.1205	0.116*	
H25C	−0.3644	0.5501	0.0210	0.116*	
S26	0.47869 (9)	0.82754 (4)	0.30872 (9)	0.0676 (3)	
O27	0.6132 (3)	0.79768 (13)	0.3588 (3)	0.0980 (9)	
O28	0.4780 (3)	0.89700 (12)	0.3260 (3)	0.1005 (9)	
O29	0.3556 (3)	0.79269 (14)	0.3249 (3)	0.1026 (9)	
C30	0.4620 (5)	0.8203 (3)	0.1534 (4)	0.1024 (14)	
F31	0.5746 (4)	0.8502 (2)	0.1247 (3)	0.1619 (13)	
F32	0.4639 (4)	0.75825 (19)	0.1220 (3)	0.1721 (14)	
F33	0.3479 (4)	0.8468 (2)	0.0881 (3)	0.1719 (15)	

### Atomic displacement parameters (Å^2^)

**Table:**

	*U*^11^	*U*^22^	*U*^33^	*U*^12^	*U*^13^	*U*^23^
C1	0.0475 (17)	0.081 (3)	0.084 (3)	0.0049 (19)	0.0198 (16)	0.009 (2)
C2	0.066 (2)	0.077 (3)	0.098 (3)	0.009 (2)	0.025 (2)	0.010 (2)
C3	0.068 (2)	0.074 (3)	0.094 (3)	−0.007 (2)	0.019 (2)	0.005 (2)
C4	0.0476 (17)	0.095 (3)	0.065 (2)	−0.013 (2)	0.0176 (15)	0.004 (2)
C5	0.053 (2)	0.092 (3)	0.086 (3)	0.016 (2)	0.0137 (18)	0.001 (2)
C6	0.077 (3)	0.082 (3)	0.108 (3)	0.027 (2)	0.016 (2)	0.007 (3)
C7	0.073 (2)	0.074 (3)	0.121 (3)	0.004 (2)	0.023 (2)	0.002 (2)
C8	0.0522 (18)	0.075 (3)	0.093 (3)	−0.0007 (19)	0.0168 (17)	0.001 (2)
C9	0.0341 (14)	0.074 (2)	0.056 (2)	0.0012 (15)	0.0129 (13)	−0.0011 (16)
N10	0.0343 (12)	0.083 (2)	0.0595 (17)	0.0019 (14)	0.0148 (11)	−0.0024 (14)
C11	0.0371 (16)	0.078 (3)	0.059 (2)	0.0031 (16)	0.0150 (14)	0.0038 (17)
C12	0.0378 (15)	0.078 (3)	0.051 (2)	−0.0011 (16)	0.0126 (13)	0.0025 (16)
C13	0.0408 (16)	0.077 (3)	0.064 (2)	−0.0013 (17)	0.0166 (14)	0.0001 (17)
C14	0.0418 (16)	0.077 (3)	0.060 (2)	0.0079 (17)	0.0122 (14)	0.0007 (17)
C15	0.0400 (16)	0.068 (2)	0.065 (2)	−0.0002 (14)	0.0159 (17)	0.0015 (17)
O16	0.0393 (10)	0.0869 (17)	0.0597 (15)	−0.0019 (10)	0.0119 (10)	−0.0009 (12)
O17	0.0402 (12)	0.157 (3)	0.0613 (15)	−0.0036 (13)	0.0155 (11)	−0.0026 (15)
C18	0.0389 (15)	0.064 (2)	0.0523 (19)	0.0018 (15)	0.0094 (13)	−0.0004 (16)
C19	0.0491 (18)	0.061 (2)	0.069 (2)	0.0097 (16)	0.0088 (15)	0.0051 (18)
C20	0.0501 (18)	0.060 (2)	0.079 (2)	0.0022 (16)	0.0066 (16)	−0.0022 (18)
C21	0.0429 (16)	0.072 (3)	0.072 (2)	0.0043 (16)	0.0035 (15)	−0.0055 (18)
C22	0.059 (2)	0.067 (3)	0.102 (3)	0.0148 (19)	−0.0019 (19)	0.004 (2)
C23	0.060 (2)	0.058 (2)	0.092 (3)	0.0033 (17)	0.0100 (18)	0.0055 (19)
Br24	0.0442 (2)	0.1080 (4)	0.1442 (5)	0.00493 (19)	−0.0065 (2)	−0.0176 (3)
C25	0.0321 (15)	0.104 (3)	0.093 (3)	0.0019 (17)	0.0103 (15)	−0.012 (2)
S26	0.0539 (5)	0.0615 (6)	0.0876 (7)	0.0009 (4)	0.0176 (4)	0.0017 (5)
O27	0.0639 (14)	0.086 (2)	0.130 (2)	0.0090 (13)	−0.0052 (14)	0.0217 (16)
O28	0.0918 (18)	0.0651 (19)	0.150 (3)	−0.0001 (13)	0.0394 (18)	−0.0211 (16)
O29	0.0775 (16)	0.098 (2)	0.144 (3)	−0.0196 (14)	0.0486 (16)	0.0098 (18)
C30	0.104 (3)	0.100 (4)	0.102 (4)	−0.001 (3)	0.020 (3)	−0.001 (3)
F31	0.162 (3)	0.227 (4)	0.115 (2)	−0.019 (3)	0.069 (2)	0.029 (2)
F32	0.250 (4)	0.141 (3)	0.120 (2)	0.006 (3)	0.034 (2)	−0.052 (2)
F33	0.139 (3)	0.201 (4)	0.134 (3)	0.005 (2)	−0.048 (2)	0.047 (2)

### Geometric parameters (Å, °)

**Table:**

C1—C2	1.342 (5)	C13—C14	1.433 (4)
C1—C11	1.429 (5)	C15—O17	1.187 (4)
C1—H1	0.9300	C15—O16	1.332 (4)
C2—C3	1.395 (5)	O16—C18	1.421 (3)
C2—H2	0.9300	C18—C19	1.361 (4)
C3—C4	1.352 (5)	C18—C23	1.367 (4)
C3—H3	0.9300	C19—C20	1.376 (4)
C4—C12	1.411 (5)	C19—H19	0.9300
C4—H4	0.9300	C20—C21	1.367 (5)
C5—C6	1.359 (5)	C20—H20	0.9300
C5—C14	1.403 (5)	C21—C22	1.372 (5)
C5—H5	0.9300	C21—Br24	1.906 (3)
C6—C7	1.403 (5)	C22—C23	1.385 (5)
C6—H6	0.9300	C22—H22	0.9300
C7—C8	1.353 (5)	C23—H23	0.9300
C7—H7	0.9300	C25—H25A	0.9600
C8—C13	1.415 (5)	C25—H25B	0.9600
C8—H8	0.9300	C25—H25C	0.9600
C9—C11	1.384 (4)	S26—O27	1.418 (2)
C9—C13	1.391 (4)	S26—O29	1.429 (2)
C9—C15	1.505 (4)	S26—O28	1.438 (3)
N10—C14	1.369 (4)	S26—C30	1.779 (5)
N10—C12	1.374 (4)	C30—F33	1.290 (5)
N10—C25	1.491 (3)	C30—F32	1.325 (5)
C11—C12	1.437 (4)	C30—F31	1.350 (5)
			
C2—C1—C11	121.5 (3)	C5—C14—C13	118.7 (3)
C2—C1—H1	119.3	O17—C15—O16	125.5 (3)
C11—C1—H1	119.3	O17—C15—C9	123.6 (3)
C1—C2—C3	120.0 (4)	O16—C15—C9	110.8 (3)
C1—C2—H2	120.0	C15—O16—C18	116.0 (2)
C3—C2—H2	120.0	C19—C18—C23	122.3 (3)
C4—C3—C2	121.7 (4)	C19—C18—O16	119.2 (3)
C4—C3—H3	119.1	C23—C18—O16	118.4 (3)
C2—C3—H3	119.1	C18—C19—C20	119.3 (3)
C3—C4—C12	120.6 (3)	C18—C19—H19	120.4
C3—C4—H4	119.7	C20—C19—H19	120.4
C12—C4—H4	119.7	C21—C20—C19	119.1 (3)
C6—C5—C14	120.0 (3)	C21—C20—H20	120.5
C6—C5—H5	120.0	C19—C20—H20	120.5
C14—C5—H5	120.0	C20—C21—C22	121.7 (3)
C5—C6—C7	122.4 (4)	C20—C21—Br24	118.6 (3)
C5—C6—H6	118.8	C22—C21—Br24	119.8 (2)
C7—C6—H6	118.8	C21—C22—C23	119.2 (3)
C8—C7—C6	118.8 (4)	C21—C22—H22	120.4
C8—C7—H7	120.6	C23—C22—H22	120.4
C6—C7—H7	120.6	C18—C23—C22	118.4 (3)
C7—C8—C13	121.6 (3)	C18—C23—H23	120.8
C7—C8—H8	119.2	C22—C23—H23	120.8
C13—C8—H8	119.2	N10—C25—H25A	109.5
C11—C9—C13	121.4 (3)	N10—C25—H25B	109.5
C11—C9—C15	120.0 (3)	H25A—C25—H25B	109.5
C13—C9—C15	118.5 (3)	N10—C25—H25C	109.5
C14—N10—C12	122.4 (2)	H25A—C25—H25C	109.5
C14—N10—C25	118.1 (3)	H25B—C25—H25C	109.5
C12—N10—C25	119.5 (3)	O27—S26—O29	115.25 (18)
C9—C11—C1	122.9 (3)	O27—S26—O28	113.86 (17)
C9—C11—C12	119.3 (3)	O29—S26—O28	116.39 (17)
C1—C11—C12	117.9 (3)	O27—S26—C30	103.3 (2)
N10—C12—C4	122.8 (3)	O29—S26—C30	102.6 (2)
N10—C12—C11	118.7 (3)	O28—S26—C30	102.8 (2)
C4—C12—C11	118.4 (3)	F33—C30—F32	107.9 (4)
C9—C13—C8	122.8 (3)	F33—C30—F31	106.0 (4)
C9—C13—C14	118.6 (3)	F32—C30—F31	107.6 (5)
C8—C13—C14	118.5 (3)	F33—C30—S26	114.7 (4)
N10—C14—C5	121.8 (3)	F32—C30—S26	110.9 (4)
N10—C14—C13	119.5 (3)	F31—C30—S26	109.4 (3)
			
C11—C1—C2—C3	−0.8 (6)	C6—C5—C14—C13	−0.8 (5)
C1—C2—C3—C4	0.4 (6)	C9—C13—C14—N10	2.0 (5)
C2—C3—C4—C12	0.8 (6)	C8—C13—C14—N10	−179.2 (3)
C14—C5—C6—C7	−0.2 (6)	C9—C13—C14—C5	−177.4 (3)
C5—C6—C7—C8	0.5 (7)	C8—C13—C14—C5	1.4 (5)
C6—C7—C8—C13	0.2 (6)	C11—C9—C15—O17	82.6 (4)
C13—C9—C11—C1	176.7 (3)	C13—C9—C15—O17	−93.9 (4)
C15—C9—C11—C1	0.3 (5)	C11—C9—C15—O16	−96.8 (3)
C13—C9—C11—C12	−2.9 (5)	C13—C9—C15—O16	86.6 (4)
C15—C9—C11—C12	−179.4 (3)	O17—C15—O16—C18	4.3 (5)
C2—C1—C11—C9	−179.7 (3)	C9—C15—O16—C18	−176.2 (3)
C2—C1—C11—C12	−0.1 (5)	C15—O16—C18—C19	85.8 (4)
C14—N10—C12—C4	−178.4 (3)	C15—O16—C18—C23	−97.4 (3)
C25—N10—C12—C4	1.3 (4)	C23—C18—C19—C20	1.0 (5)
C14—N10—C12—C11	−0.6 (4)	O16—C18—C19—C20	177.7 (3)
C25—N10—C12—C11	179.1 (3)	C18—C19—C20—C21	−1.0 (5)
C3—C4—C12—N10	176.1 (3)	C19—C20—C21—C22	0.0 (5)
C3—C4—C12—C11	−1.7 (5)	C19—C20—C21—Br24	−179.7 (3)
C9—C11—C12—N10	3.0 (4)	C20—C21—C22—C23	0.9 (6)
C1—C11—C12—N10	−176.6 (3)	Br24—C21—C22—C23	−179.4 (3)
C9—C11—C12—C4	−179.1 (3)	C19—C18—C23—C22	−0.1 (5)
C1—C11—C12—C4	1.3 (4)	O16—C18—C23—C22	−176.8 (3)
C11—C9—C13—C8	−178.3 (3)	C21—C22—C23—C18	−0.9 (6)
C15—C9—C13—C8	−1.8 (5)	O27—S26—C30—F33	−177.5 (4)
C11—C9—C13—C14	0.5 (5)	O29—S26—C30—F33	62.4 (4)
C15—C9—C13—C14	176.9 (3)	O28—S26—C30—F33	−58.8 (4)
C7—C8—C13—C9	177.7 (4)	O27—S26—C30—F32	60.0 (4)
C7—C8—C13—C14	−1.1 (5)	O29—S26—C30—F32	−60.2 (4)
C12—N10—C14—C5	177.5 (3)	O28—S26—C30—F32	178.7 (4)
C25—N10—C14—C5	−2.2 (5)	O27—S26—C30—F31	−58.5 (4)
C12—N10—C14—C13	−1.9 (5)	O29—S26—C30—F31	−178.6 (3)
C25—N10—C14—C13	178.4 (3)	O28—S26—C30—F31	60.2 (4)
C6—C5—C14—N10	179.8 (3)		

### Hydrogen-bond geometry (Å, °)

**Table:**

*Cg*4 is the centroid of the C18–C23 ring.

**Table:**

*D*—H···*A*	*D*—H	H···*A*	*D*···*A*	*D*—H···*A*
C2—H2···O27^i^	0.93	2.59	3.361 (5)	141
C4—H4···O28^ii^	0.93	2.50	3.365 (4)	155
C20—H20···O27	0.93	2.50	3.176 (4)	130
C25—H25A···*Cg*4^iii^	0.96	2.81	3.569 (4)	136
C25—H25B···O28^ii^	0.96	2.53	3.472 (5)	167

Symmetry codes:  (i) −*x*+1, *y*−1/2, −*z*+1/2; (ii) −*x*, *y*−1/2, −*z*+1/2; (iii) *x*−1, *y*, *z*.

### Table 2 C–Br···π and C–F···π interactions (Å,°).

**Table:**

*X*	*I*	*J*	*I*···*J*	*X*···*J*	*X*–*I*···*J*
C21	Br24	*Cg*1^iv^	3.958 (2)	4.158 (3)	82.3 (1)
C21	Br24	*Cg*3^iv^	3.937 (2)	4.235 (4)	85.4 (2)
C30	F31	*Cg*4^v^	3.212 (4)	4.305 (5)	137.5 (3)

Symmetry codes: (iv) *x* + 1, *y*, *z*; (v) *x*, –*y* + 3/2, *z* – 1/2.Notes: *Cg*1, *Cg*3 and *Cg*4 are the centroids of the C9/N10/C11–C14, C5–C8/C13/C14 and C18–C23 rings, respectively.

### Table 3 π–π interactions (Å,°).

**Table:**

*I*	*J*	*CgI*···*CgJ*	Dihedral angle	*CgI*_Perp	Cg*I*_Offset
1	2^vi^	3.650 (2)	2.82 (16)	3.623 (2)	0.444 (2)
2	1^vi^	3.650 (2)	2.82 (16)	3.623 (2)	0.444 (2)

Symmetry code: (vi) –*x*, –*y* + 1, –*z*.Notes: *Cg*1 and *Cg*2 are the centroids of the C9/N10/C11–C14 and C1–C4/C11/C12 rings, respectively. Cg*I*···Cg*J* is the distance between ring centroids. The dihedral angle is that between the planes of the rings *I* and *J*. *CgI*_Perp is the perpendicular distance of *CgI* from ring *J*. Cg*I*_Offset is the distance between *CgI* and perpendicular projection of *CgJ* on ring *I*.
